# Risk Factors Associated with the Community-Acquired Colonization of Extended-Spectrum Beta-Lactamase (ESBL) Positive *Escherichia Coli.* An Exploratory Case-Control Study

**DOI:** 10.1371/journal.pone.0074323

**Published:** 2013-09-11

**Authors:** Rasmus Leistner, Elisabeth Meyer, Petra Gastmeier, Yvonne Pfeifer, Christoph Eller, Petra Dem, Frank Schwab

**Affiliations:** 1 Institute of Hygiene and Environmental Medicine, Charité University Medicine Berlin, Berlin, Germany; 2 German National Reference Center for the Surveillance of Nosocomial Infections, Berlin, Germany; 3 Robert Koch Institute, Wernigerode, Germany; Amphia Ziekenhuis, Netherlands

## Abstract

**Background:**

The number of extended-spectrum beta-lactamase (ESBL) positive (+) *Escherichia coli* is increasing worldwide. In contrast with many other multidrug-resistant bacteria, it is suspected that they predominantly spread within the community. The objective of this study was to assess factors associated with community-acquired colonization of ESBL (+) *E. coli*.

**Methods:**

We performed a matched case-control study at the Charité University Hospital Berlin between May 2011 and January 2012. Cases were defined as patients colonized with community-acquired ESBL (+) *E. coli* identified <72 h after hospital admission. Controls were patients that carried no ESBL-positive bacteria but an ESBL-negative *E.coli* identified <72 h after hospital admission. Two controls per case were chosen from potential controls according to admission date. Case and control patients completed a questionnaire assessing nutritional habits, travel habits, household situation and language most commonly spoken at home (mother tongue). An additional rectal swab was obtained together with the questionnaire to verify colonization status. Genotypes of ESBL (+) *E. coli* strains were determined by PCR and sequencing. Risk factors associated with ESBL (+) *E. coli* colonization were analyzed by a multivariable conditional logistic regression analysis.

**Results:**

We analyzed 85 cases and 170 controls, respectively. In the multivariable analysis, speaking an Asian language most commonly at home (OR = 13.4, CI 95% 3.3–53.8; p<0.001) and frequently eating pork (≥3 meals per week) showed to be independently associated with ESBL colonization (OR = 3.5, CI 95% 1.8–6.6; p<0.001). The most common ESBL genotypes were CTX-M-1 with 44% (n = 37), CTX-M-15 with 28% (n = 24) and CTX-M-14 with 13% (n = 11).

**Conclusion:**

An Asian mother tongue and frequently consuming certain types of meat like pork can be independently associated with the colonization of ESBL-positive bacteria. We found neither frequent consumption of poultry nor previous use of antibiotics to be associated with ESBL colonization.

## Introduction

The prevalence of 3^rd^ generation cephalosporin resistance among gram negative bacteria has increased worldwide in recent years [1]. The predominant resistance mechanism is the production of extended-spectrum beta-lactamase (ESBL). In contrast with other multidrug-resistant bacteria, it is suspected that the ESBL-mediated resistance has been spreading mainly throughout the community and not primarily within healthcare-related institutions. Travel to endemic countries is an often-discussed risk factor for ESBL colonization [2], [3]. Another basis for discussion are the results of recent studies that have shown frequent ESBL colonization in poultry. This suggests spread through the food chain [4], [5], [6], [7]. To assess these factors we performed a case-control study comparing people who acquired ESBL-positive *Escherichia coli* in the community and people who do not carry ESBL-positive bacteria.

## Materials and Methods

### Ethics Statement

A confirmatory ethics vote was obtained from the Charité University Medicine ethics committee (processing number EA4/022/11). Following the Charité University Medicine ethics committee, study subjects had to be at least 18 years of age and were recruited from the Charité University Hospital. Written informed consent was obtained from all subjects. We categorized subjects into cases (colonized with ESBL-positive *E. coli*) and controls (not colonized with an ESBL positive organism but colonized with ESBL negative *E. coli*) irrespective of any socially constructed grouping (e.g. race/ethnicity, age, disease/disabilities, religion, sex/gender or sexual orientation).

### Setting, Study Design and Questionnaire

The case control study was performed at the Charité University Hospital in Berlin, Germany, a tertiary care university hospital with over 120,000 inpatients per year. The study was designed to detect an odds ratio greater than or equal to 4 with a significance level of 0.05 and a power of 0.80. According to the ECDC report on antimicrobial resistance in Europe, we assumed an ESBL colonization rate of 6% [8]. Based on these requirements 79 cases and 158 controls should be observed. Cases and controls were selected from the Charité patient population in a study period of nine months (May 2011 to January 2012). Patients included were interviewed by our study staff members who obtained informed consent, a completed study questionnaire and a rectal swab. The study questionnaire enquired the patients’ age, sex, body mass index (BMI), living situation (alone, shared apartment with a vegetarian/meat eater) and animal contact. Furthermore, we assessed healthcare-related risk factors like antimicrobial therapy and hospital stay, and further related risk factors like urinary tract infections, diarrhea (each within the last 12 months) and colonization with methicillin-resistant *S. aureus* (MRSA), vancomycin resistant *Enterococci* (VRE) or *Clostridium difficile* within the prior 12 months [9], [10], [11]. We also included the patients’ travel destinations within the last 12 months, as well as the most commonly spoken language in the patients’ home. For easier reference in this article we refer to this parameter as the patients’ mother tongue. Dietary habits were estimated by assessing the average number of meals with beef, pork, poultry and fish meals per week within the last 12 months. Except underlying co-morbidities, all patient parameters were assessed through the interview questionnaire. As a measurement for underlying co-morbidities, the Charlson co-morbidity index (CCI) was obtained by analyzing the patients’ electronic files.

### Cases and Controls

Cases and controls had to be at least 18 years of age. They were found by search of the database of the Charité department of microbiology. Cases were defined as patients that carried ESBL -positive *E. coli* (confirmed ESBL phenotype) and controls were defined as patients that carried no ESBL-positive bacteria but an ESBL-negative *E.coli.* Both were found in a clinically obtained specimen within the first 72 hours after admission. We considered all types of specimen (urine culture, blood culture, rectal swab, stool sample etc.) that were sent to the microbiology laboratory within the study period for microbiological examination to be clinically obtained specimens. All potential cases and controls that were colonized with an ESBL-positive bacterium in a previous hospital stay within the last 12 months were excluded. Each case was matched to two controls. In order to verify the colonization status at the time of enquiry, we obtained an additional rectal swab at the interview (verification swab). The study was approved in advance by the Charité ethics commission.

### Microbiological Methods

For initial identification of cases and controls, bacterial strains were tested using the Vitek 2 automated system. Susceptibility testing of ESBL-positive bacteria was performed as minimal inhibitory concentration (MIC) and included testing of cefotaxim, ceftriaxon and ceftazidime. Confirmation of ESBL production was performed by a MIC dilution test on a multi-well microtiter plate. 3^rd^ generation cephalosporins were tested alone and in combination with clavulanic acid. All verification swabs were inoculated onto chrom ID ESBL agar (BioMerieux). ESBL-positive isolates were affirmed by Double Disc Synergie Testing using 3rd generation cephalosporins with/without clavulanic acid (Mast). Species confirmation was done by API20E (BioMerieux). All *E. coli* isolates with confirmed ESBL phenotype were further analyzed by the Robert Koch Institute, Wernigerode, Germany. Antimicrobial susceptibilities to 12 different antibiotics (ampicillin, cefotaxime, ceftazidime, cefoxitin, nalidixic acid, ciprofloxacin, gentamicin, amikacin, streptomycin, chloramphenicol, tetracycline, and trimethoprim-sulfamethoxazole) were performed according to CLSI criteria [12]. Presence of beta-lactamase genes was tested by PCR amplification and sequencing of ESBL genes *bla*_TEM_, *bla*_SHV_, *bla*_CTX-M_ [13], [14]. In isolates with minimum inhibitory concentrations (MICs) for ciprofloxacin (MIC_CIP_ = 0.125– >64 mg/L), the presence of plasmid-mediated quinolone resistance (PMQR) genes (*qnr*-type genes, *aac(6′)1b-cr*) was determined [7], [14]. *E. coli* phylogenetic groups were identified by a previously described PCR-based method [15].

### Statistical Methods

Categorical variables were compared and tested using the Chi-square test. Continuous variables were compared and tested using the Wilcoxon rank sum test. A multivariable analysis was performed to estimate the effects of factors independently associated with ESBL-positive *E. coli* colonization using a stepwise forward conditional logistic regression. The p-values for including a variable in the model was 0.05 and for excluding 0.06 respectively. Odds ratios (OR) and their 95% confidence intervals (CI 95%) were calculated. SPSS (IBM SPSS statistics, Somer, NY, USA) and SAS (SAS Institute, Cary, NC, USA) were used for these analyses.

## Results

**Figure 1:**
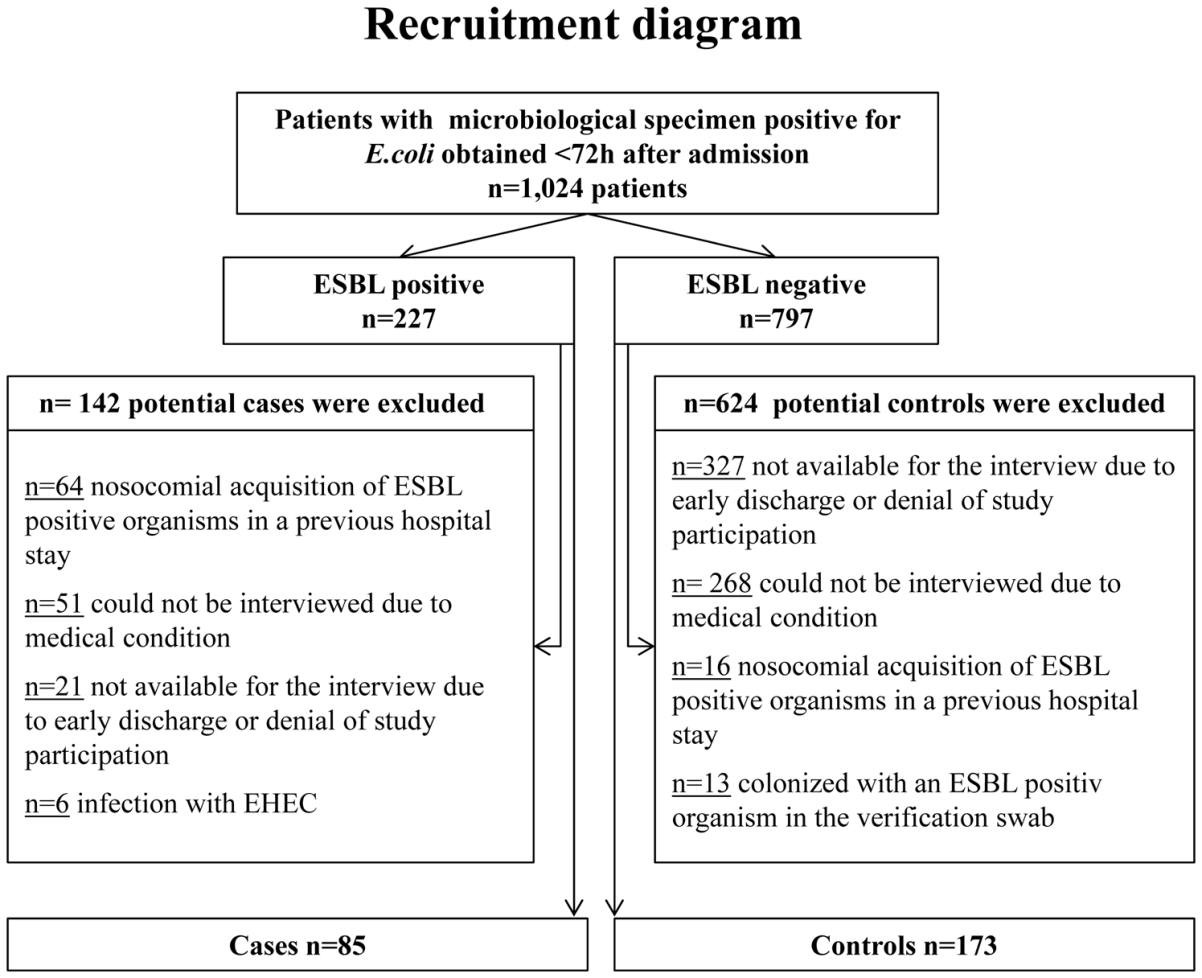
Recruitment diagram of cases and controls. EHEC, enterohemorrhagic *E. coli.* ESBL, extendend-spectrum beta-lactamase.

We collected data on all consecutive cases colonized with ESBL positive-*E.coli* during the study period, altogether 227 patients. Sixty-four patients had to be excluded due to prior nosocomial acquisition of ESBL, 51 patients could not be interviewed due to medical condition (intubation, dementia, fatal condition), and 21 patients were not available for the interview due to early discharge or denial of study participation. Six patients were furthermore excluded because they carried an ESBL-positive enterohemorrhagic *E.coli* (EHEC) and were part of the EHEC outbreak that took place in Germany during the study period [16]. Eighty-five patients met our definition of community-acquired ESBL-positive *E.coli* and were eligible. Seven-hundred ninety-seven potential controls were eligible for further individual screening and one-hundred seventy-three controls were finally available as controls. We matched two controls to one case. If more than two controls were eligible, we selected the control with the closest admission date to the respective case admission date. A flow diagram documenting the recruitment process for cases and controls is displayed in Figure 1. The analysis of base line parameter for included and excluded patients is shown in Table 1. There were no differences in the baseline parameters between cases and controls (Table 2). The results of the questionnaire based interview are shown in Table 3. None of the included patients lived on a vegan or vegetarian diet. There was neither difference in the living situation nor in the animal contact or health care-related risk factors between of cases and controls. In the multivariable conditional logistic regression analysis for ESBL colonization, Asian mother tongue (Odds ratio 13.4, IC 95% 3.3–53.8, p-value <0.001) and frequent consumption of pork (Odds ratio 3.5, CI 95% 1.8–6.6, p-value <0.001) were independently associated with the colonization of ESBL-positive *E.* coli (Table 4). Of the 11 case patients with Asian mother tongue, only one consumed pork. This person was also among the group of frequent pork meat consumers and had an Eastern Asian mother tongue. To identify cases of household transmission, we controlled these 11 patients for familiar relations by surname and postal code. No similarities were found. Among the 50 patients with frequent pork meat consumption, 96% (n = 48) gave German as their mother tongue.

**Table 1:** Comparison of basic parameters of excluded and included cases, controls and overall considered patients.

	Cases				Controls		
Parameter	Excluded (n = 142)	Included (n = 85)		P-value	Excluded (n = 624)	Included (n = 173)	P-value
Median Age (IQR)	66 (52; 74)	62 (53; 72)		0.209	68 (53; 76)	67 (55; 73)	0.341^a^
Median CCI (IQR)	5 (2; 7)	5 (3; 7)		0.832	5 (2; 8)	4 (2; 6)	**0.048^a^**
Male sex, n (%)	76 (54)	42 (49)		0.585	232 (37)	72 (42)	0.165
**Overall included and excluded patients**							
**Parameter**	**Excluded patients (n = 766)**		**Included patients (n = 258)**		**P-value**		
Median Age (IQR)	67 (53; 75)		66 (53; 72)		p = 0.085^ a^		
Median CCI (IQR)	5 (2; 8)		4 (3; 7)		p = 0.112^ a^		
Male sex n (%)	n = 308 (40%)		n = 114 (44%)		p = 0.273		

IQR, interquartile range. CCI, Charlson co-morbidity Index. P-value from Chi-square test or^ a^Wilcoxon rank sum test, respectively.

**Table 2:** Demographic data for included cases colonized with ESBL-positive *E. coli* and controls without ESBL colonization.

Parameter	Cases (N = 85) n (%)	Controls (N = 170) n (%)	P-value
Male	41 (48.2%)	72 (42.4%)	0.372
Median Age (IQR)	63 (53;72)	63 (56;73)	0.482^a^
Median BMI (IQR)	26 (22;29)	26 (22;29)	0.341^a^
Median CCI (IQR)	4 (2;6)	0 (2;7)	0.581^a^
Myocardial infarction	18 (10.6%)	9 (10.6%)	1.000
Congestive heart failure	21 (12.4%)	9 (10.6%)	0.680
Peripheral vascular disease	12 (7.1%)	7 (8.2%)	0.736
Cerebrovascular disease	4 (2.4%)	1 (1.2%)	0.523
Dementia	1 (0.6%)	0 (0.0%)	0.479
Chronic lung disease	24 (14.1%)	14 (16.5%)	0.619
Rheuma	4 (2.4%)	5 (5.9%)	0.150
Peptic ulcer	7 (4.1%)	2 (2.4%)	0.472
Mild liver disease	13 (7.6%)	4 (4.7%)	0.375
Diabetes without complication	26 (15.3%)	16 (18.8%)	0.474
Diabetes with chronic complication	9 (5.3%)	6 (7.1%)	0.572
Renal disease	52 (30.6%)	28 (32.9%)	0.703
Cancer	33 (19.4%)	15 (17.6%)	0.734
Moderate to severe liver disease	2 (1.2%)	1 (1.2%)	1.000
Metastatic cancer	20 (11.8%)	15 (17.6%)	0.198
AIDS	1 (0.6%)	0 (0.0%)	0.479
Hemiplegia	4 (2.4%)	1 (1.2%)	0.523
Leukemia	2 (1.2%)	2 (2.4%)	0.476
Lymphoma	8 (9.4%)	12 (14.1%)	0.680

IQR, interquartile range. BMI, Body Mass Index. CCI, Charlson co-morbidity Index. AIDS, acquired immune deficiency syndrome. P-value from Chi-square test or^ a^Wilcoxon rank sum test, respectively.

**Table 3:** Results of the questionnaire based interview: cases colonized with ESBL-positive *E. coli* and controls without ESBL colonization.

Parameter	Cases (N = 85) n (%)		Controls (N = 170) n (%)	P-value
*Mother tongue (most commonly spoken language)*				
German	73(86%)		157(92%)	0.101
Northern/Central European	0		2(1%)	0.315
Southern European	1(1%)		1(1%)	0.616
Eastern European	0		6(4%)	0.080
Asian	11(13%)		4(2%)	0.001
*Meat consumption ≥3 meals per week*				
Beef	1(1%)		13(8%)	0.032
Veal	1(1%)		2(1%)	1.000
Pork	50 (59%)		67(39%)	0.003
Poultry	21(25%)		37(22%)	0.597
Fish	10 (12%)		18(11%)	0.777
*Travel destinations within the last 12 months*				
Europe	14 (17%)		34 (20%)	0.497
Asia	11 (13%)		11 (7%)	0.083
East Asia	1 (1%)		0	0.156
West Asia	9 (11%)		8 (5%)	0.076
South Asia	1 (1%)		0	0.156
North America	1 (1%)		2 (1%)	1.000
South America	1 (1%)		0	0.156
Africa	1 (1%)		4 (2%)	0.523
Australia/New Zealand	0		0	–
*Health care related risk factors and carriage of other MDROs* *within prior 12 months*				
Hospital stay		67 (79%)	118 (69%)	0.112
Antimicrobial therapy		76 (89%)	140 (82%)	0.140
Diarrhea		45 (53%)	61 (36%)	0.009
Urinary tract infection		37 (44%)	95 (56%)	0.063
MRSA		3 (3.5%)	2 (1.2%)	0.201
VRE		2 (2.4%)	1 (0.6%)	0.218
Clostridium difficile		1 (1.2%)	1 (0.6%)	0.616
*Living situation*				
Alone		26 (31%)	59 (35%)	0.511
With meat eater		59 (69%)	110 (65%)	0.454
Only with vegetarians		0	0	–
In a long term care facility		0	1 (0.6%)	0.479
*Animal contact*				
Domestic animals		29 (34.1%)	60 (35.3%)	0.853
Farm animals		5 (0.6%)	14 (0.8%)	0.500

MRSA, Methicillin resistant *S.aureus*; VRE, Vancomycin resistant *Enterococcus.* MDRO, multi drug resistant organism. P-value from Chi-square test. East Asia: China, Japan, Mongolia, North Korea, South Korea, Taiwan. West Asia: Bahrain, Iraq, Israel, Yemen, Jordan, Qatar, Kuwait, Lebanon, Oman, Palestine, Saudi Arabia, Syria, Turkey, UAE, Cyprus. South Asia: Afghanistan, Bangladesh, Bhutan, India, Iran, Maldives, Nepal, Pakistan, Sri Lanka.

**Table 4:** Results of the multivariable conditional logistic regression analysis of risk factors for colonization with ESBL-positive *E. coli*.

Associated factor	Odds Ratio	95% CI	P-value
Asian mother tongue	13.4	3.3–53.8	<0.001
Frequent consumption of pork	3.5	1.8–6.6	<0.001

**Figure 2:**
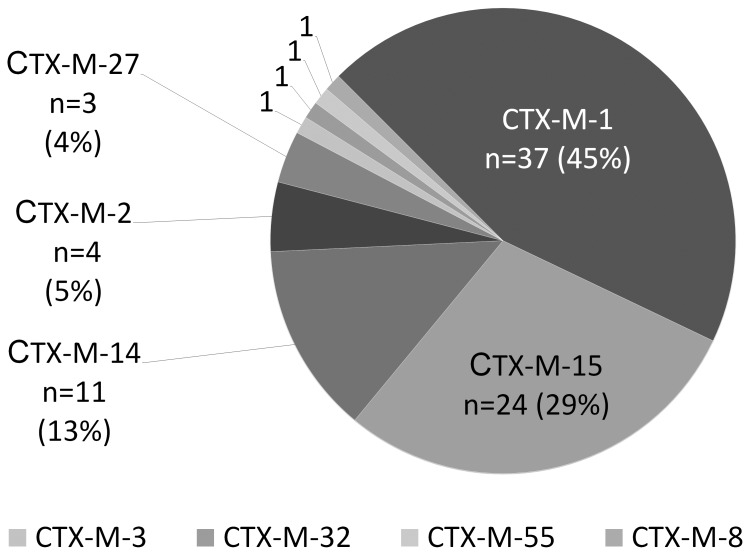
Distribution of CTX-M-genotypes in n = 83 CTX-M-positive community-acquired ESBL *E. coli* isolates.

Molecular analyses revealed CTX-M-type ESBL in 83 *E. coli* isolates (98%); the two remaining isolated were positive for ESBL-types TEM-52 and SHV-12, respectively. The most common CTX-M genotypes were CTX-M-1 with 45% (n = 37), CTX-M-15 with 29% (n = 24) and CTX-M-14 with 13% (n = 11). CTX-M-2 was identified in 4 *E. coli*, CTX-M-27 in 3 *E. coli* and further variants (CTX-M-3, CTX-M-8, CTX-M-32, CTX-M-55) were identified in single isolates. The distribution of the CTX-M genotypes is shown in Figure 2. Twenty-three isolates were additionally positive for beta-lactamase TEM-1. Furthermore, in 16 isolates with increased MIC for ciprofloxacin the PMQR gene *aac(6′)1b-cr* was identified. The 85 *E. coli* were assigned to the four phylogenic groups B2 (40%; n = 34), B1 (25%; n = 21), D (19%; n = 16) and A (16%; n = 14). Additionally, we found that 67% of the CTX-M-15-positive *E. coli* belonged to phylogenetic group B2, in contrast with the CTX-M-1 positive strains that were assigned to the four phylogenetic groups in nearly equal proportions (21–30%).

## Discussion

In the present study, an Asian mother tongue and frequent consumption of pork were independently associated with the acquisition of ESBL-positive *E. coli* in the community. ESBL-positive organisms are widespread especially in some Asian countries [1]. ESBL prevalence in Asia ranges between 13% and 79% [17]. Recent studies have shown that returning travelers and patients from high endemic countries are at increased risk for colonization with ESBL-positive Enterobacteriaceae. Tangden et al. examined healthy Sweden-based travelers and found a significant increase in ESBL colonization after returning from Asian countries [3]. This effect may be epidemiologically of great importance. A study from Switzerland showed that the transmission of ESBL-positive bacteria within the community significantly exceeds nosocomial transmission rates [14]. Wickramasinghe et al. analyzed stool samples from general practices and outpatients in the UK and found significantly higher prevalence in patients with Middle Eastern or South Asian background [18]. Our findings support these results and show that an Asian family background may yield an increased possibility of ESBL acquisition.

Recently, human colonization with ESBL-positive organisms through the food chain has been widely discussed [4], [7], [19], [20]. Most studies concerning ESBL-colonized meat found ESBL organisms in living poultry or chicken meat. Studies from the Netherlands even found significant similarities in ESBL genes in strains from poultry and humans [5], [6], [7]. In a recent ESBL-screening of retail chicken meat from German supermarkets located in the same area as our study location, CTX-M-1 and SHV-12 were found to be the predominant ESBL genotypes [4]. However, to our knowledge, there has yet not been an experimental study that verified the possibility of colonization through the consumption of contaminated food. Even though the consumption of pork meat but not the consumption of poultry meat was associated with ESBL colonization in this study our result supports the possible transmission of ESBL-positive bacteria through the food chain. The most commonly detected ESBL genotype in European pigs is CTX-M-1 and was also the predominant genotype in our study isolates [19].

Interestingly, Overdevest et al. found mostly CTX-M-1 in human isolates from the community but CTX-M-14 in clinical blood culture isolates [6], [7]. Other studies analyzed patients without healthcare contact in the previous 1 to 3 months [21], [22], [23]. The ESBL genotypes they found were mainly CTX-M-14 and CTX-M-15, and the detected risk factors for colonization in these studies were recent hospital admission and use of antibiotics. The risk factors found indicate that these studies assessed cases of healthcare-acquired ESBL colonization. The difference between the healthcare-acquired genotypes and genotypes circling in the community might be associated with the pressure deriving from the antibiotics used in the hospital [22]. Ceftazidime e.g. is a substrate for CTX-M-15 but not for CTX-M-1 [24], [25]. Studies on duration of ESBL colonization showed that ESBL-positive patients can be colonized for over 6 months [26], [27], [28]. In order to analyze acquisition in the community, only patients with a rather long period after their most recent positive test for ESBL should be included. We took this into account and analyzed a patient population that had not been detected positive for ESBL-producing bacteria for at least 12 months. Most patients in this study had been hospitalized in the previous year. However, the most commonly reported factors associated with healthcare-acquired ESBL colonization were not independently associated with ESBL colonization in this study.

### Limitations

This is a questionnaire-based study. Bias associated with this kind of studies may result in unanticipated communication barriers between the investigator and the respondents that yield inaccurate results [29]. Possible bias in study design would lead to e.g. recall bias, subjective perception of the interviewed patient or influence of the interviewer. In order to minimize the risk of questionnaire bias, we concentrated on short and simple questions avoiding difficult or technical jargon. Nevertheless, some questions concern rather complicated information like travel history or diet habits. We therefore decided to perform a personal interview. However, the generalizability of our results might be limited since the analyzed cohort consisted of a selected population: hospitalized patients with *E. coli* identified in clinical cultures obtained within the first 72 hours of admission that did not fulfill any of the exclusion criteria. Even though we excluded all patients with known former ESBL colonization, we did not assess the previous colonization status of all patients. Since many of the included cases have been hospitalized previously, the possibility of a community-onset healthcare-associated infection cannot be excluded. We controlled for common risk factors associated with healthcare-acquired ESBL colonization like prior hospital stay, antimicrobial therapy, urinary tract infection and co-colonization with other MDROs. Nevertheless we did not completely adjust for all associated factors such as the previous application of a central venous catheter. Due to the known ESBL colonization status and related single room treatment an interviewer or interviewee blinding was not realizable. We did not assess whether the patients were infected or colonized with the respective pathogen. These limitations may have introduced assessment bias and may herewith have accounted for the observed associations between carriage of ESBL and Asian background or consumption of meat.

### Conclusion

In this exploratory case control study, colonization with ESBL positive *E. coli* was mor commonly found in patients with an Asian mother tongue. Frequent consumption of pork meat was another factor independently associated with the colonization of ESBL-positive *E. coli*. The mostly found ESBL genotype in the community was CTX-M-1. Further confirmatory studies are needed to prove these results.

## References

[1] ECDC (2011) Annual epidemiological report. European Centre for Disease Prevention and Control (ECDC), Stockholm, Sweden Reporting on 2009 surveillance data and 2010 epidemic intelligence data. Reporting on 2009 surveillance data and 2010 epidemic intelligence data: 183–185.

[2] Meyer E, Gastmeier P, Kola A, Schwab F (2012) Pet animals and foreign travel are risk factors for colonisation with extended-spectrum beta-lactamase-producing Escherichia coli. Infection 40: 685–687. 22971936 10.1007/s15010-012-0324-8

[3] Tangden T, Cars O, Melhus A, Lowdin E (2010) Foreign travel is a major risk factor for colonization with Escherichia coli producing CTX-M-type extended-spectrum beta-lactamases: a prospective study with Swedish volunteers. Antimicrob Agents Chemother 54: 3564–3568. 20547788 10.1128/AAC.00220-10 PMC2934993

[4] Kola A, Kohler C, Pfeifer Y, Schwab F, Kuhn K, et al (2012) High prevalence of extended-spectrum-beta-lactamase-producing Enterobacteriaceae in organic and conventional retail chicken meat, Germany. J Antimicrob Chemother 67: 2631–2634. 22868643 10.1093/jac/dks295

[5] Leverstein-van Hall MA, Dierikx CM, Cohen Stuart J, Voets GM, van den Munckhof MP, et al (2011) Dutch patients, retail chicken meat and poultry share the same ESBL genes, plasmids and strains. Clin Microbiol Infect 17: 873–880. 21463397 10.1111/j.1469-0691.2011.03497.x

[6] Overdevest I, Willemsen I, Rijnsburger M, Eustace A, Xu L, et al (2011) Extended-spectrum beta-lactamase genes of Escherichia coli in chicken meat and humans, The Netherlands. Emerg Infect Dis 17: 1216–1222. 21762575 10.3201/eid1707.110209 PMC3381403

[7] Kluytmans JA, Overdevest IT, Willemsen I, Kluytmans-van den Bergh MF, van der Zwaluw K, et al.. (2012) Extended-Spectrum beta-Lactamase-Producing Escherichia coli From Retail Chicken Meat and Humans: Comparison of Strains, Plasmids, Resistance Genes, and Virulence Factors. Clin Infect Dis doi:101093/cid/cis929. 10.1093/cid/cis929 23243181

[8] European Center for Disease Prevention and Control (ECDC) (2011) Antimicrobial resistance surveillance in Europe 2010. Annual Report of the European Antimicrobial Resistance Surveillance Network (EARS-Net). Available: http://ecdc.europa.eu/en/healthtopics/antimicrobial_resistance/epidemiological_data/Pages/ears-net_annual_reports.aspx.

[9] Enoch DA, Brown F, Sismey AW, Mlangeni DA, Curran MD, et al (2012) Epidemiology of extended-spectrum beta-lactamase-producing Enterobacteriaceae in a UK district hospital; an observational study. J Hosp Infect 81: 270–277. 22742987 10.1016/j.jhin.2012.05.006

[10] Doernberg SB, Winston LG (2012) Risk factors for acquisition of extended-spectrum beta-lactamase-producing Escherichia coli in an urban county hospital. Am J Infect Control 40: 123–127. 21775020 10.1016/j.ajic.2011.04.001

[11] Meyer E, Ziegler R, Mattner F, Schwab F, Gastmeier P, et al (2011) Increase of patients co-colonised or co-infected with methicillin-resistant Staphylococcus aureus, vancomycin-resistant Enterococcus faecium or extended-spectrum beta-lactamase-producing Enterobacteriaceae. Infection 39: 501–506. 21710119 10.1007/s15010-011-0154-0

[12] CLSI (2012) Performance Standards for Antimicrobial Susceptibility Testing. Clinical and Laboratorical Sstandards Institute (CLSI), Wayne, PA, USA. Twenty-second Informational Supplement M100-S22: 1–184.

[13] Grobner S, Linke D, Schutz W, Fladerer C, Madlung J, et al (2009) Emergence of carbapenem-non-susceptible extended-spectrum beta-lactamase-producing Klebsiella pneumoniae isolates at the university hospital of Tubingen, Germany. J Med Microbiol 58: 912–922. 19502377 10.1099/jmm.0.005850-0

[14] Hilty M, Betsch BY, Bogli-Stuber K, Heiniger N, Stadler M, et al (2012) Transmission dynamics of extended-spectrum beta-lactamase-producing Enterobacteriaceae in the tertiary care hospital and the household setting. Clin Infect Dis 55: 967–975. 22718774 10.1093/cid/cis581 PMC3436924

[15] Clermont O, Bonacorsi S, Bingen E (2000) Rapid and simple determination of the Escherichia coli phylogenetic group. Appl Environ Microbiol 66: 4555–4558. 11010916 10.1128/aem.66.10.4555-4558.2000 PMC92342

[16] Frank C, Werber D, Cramer JP, Askar M, Faber M, et al (2011) Epidemic profile of Shiga-toxin-producing Escherichia coli O104:H4 outbreak in Germany. N Engl J Med 365: 1771–1780. 21696328 10.1056/NEJMoa1106483

[17] Hawser SP, Bouchillon SK, Hoban DJ, Badal RE, Hsueh PR, et al (2009) Emergence of high levels of extended-spectrum-beta-lactamase-producing gram-negative bacilli in the Asia-Pacific region: data from the Study for Monitoring Antimicrobial Resistance Trends (SMART) program, 2007. Antimicrob Agents Chemother 53: 3280–3284. 19506060 10.1128/AAC.00426-09 PMC2715591

[18] Wickramasinghe NH, Xu L, Eustace A, Shabir S, Saluja T, et al (2012) High community faecal carriage rates of CTX-M ESBL-producing Escherichia coli in a specific population group in Birmingham, UK. J Antimicrob Chemother 67: 1108–1113. 22403261 10.1093/jac/dks018

[19] Ewers C, Bethe A, Semmler T, Guenther S, Wieler LH (2012) Extended-spectrum beta-lactamase-producing and AmpC-producing Escherichia coli from livestock and companion animals, and their putative impact on public health: a global perspective. Clin Microbiol Infect 18: 646–655. 22519858 10.1111/j.1469-0691.2012.03850.x

[20] Tian GB, Wang HN, Zhang AY, Zhang Y, Fan WQ, et al (2012) Detection of clinically important beta-lactamases in commensal Escherichia coli of human and swine origin in western China. J Med Microbiol 61: 233–238. 21940649 10.1099/jmm.0.036806-0

[21] Han JH, Kasahara K, Edelstein PH, Bilker WB, Lautenbach E (2012) Risk Factors for Infection or Colonization with CTX-M Extended-Spectrum-beta-Lactamase-Positive Escherichia coli. Antimicrob Agents Chemother 56: 5575–5580. 22890772 10.1128/AAC.01136-12 PMC3486585

[22] Nicolas-Chanoine MH, Gruson C, Bialek-Davenet S, Bertrand X, Thomas-Jean F, et al.. (2012) 10-Fold increase (2006–11) in the rate of healthy subjects with extended-spectrum beta-lactamase-producing Escherichia coli faecal carriage in a Parisian check-up centre. J Antimicrob Chemother doi:101093/jac/dks429. 10.1093/jac/dks429 23143897

[23] Rodriguez-Bano J, Navarro MD, Romero L, Martinez-Martinez L, Muniain MA, et al (2004) Epidemiology and clinical features of infections caused by extended-spectrum beta-lactamase-producing Escherichia coli in nonhospitalized patients. J Clin Microbiol 42: 1089–1094. 15004058 10.1128/JCM.42.3.1089-1094.2004 PMC356843

[24] Tzouvelekis LS, Tzelepi E, Tassios PT, Legakis NJ (2000) CTX-M-type beta-lactamases: an emerging group of extended-spectrum enzymes. Int J Antimicrob Agents 14: 137–142. 10720804 10.1016/s0924-8579(99)00165-x

[25] Baraniak A, Fiett J, Hryniewicz W, Nordmann P, Gniadkowski M (2002) Ceftazidime-hydrolysing CTX-M-15 extended-spectrum beta-lactamase (ESBL) in Poland. J Antimicrob Chemother 50: 393–396. 12205064 10.1093/jac/dkf151

[26] Birgand G, Armand-Lefevre L, Lolom I, Ruppe E, Andremont A, et al.. (2012) Duration of colonization by extended-spectrum beta-lactamase-producing Enterobacteriaceae after hospital discharge. Am J Infect Control doi: 10.1016/j.ajic.2012.05.015. 10.1016/j.ajic.2012.05.015 22998785

[27] Li B, Zhong Y, Fu XC, Qiu YH, Wang SY, et al (2012) Duration of stool colonization in healthy medical students with extended-spectrum-beta-lactamase-producing Escherichia coli. Antimicrob Agents Chemother 56: 4558–4559. 22687506 10.1128/AAC.00171-12 PMC3421574

[28] Tham J, Walder M, Melander E, Odenholt I (2012) Duration of colonization with extended-spectrum beta-lactamase-producing Escherichia coli in patients with travellers’ diarrhoea. Scand J Infect Dis 44: 573–577. 22292796 10.3109/00365548.2011.653582

[29] Choi BC, Pak AW (2005) A catalog of biases in questionnaires. Prev Chronic Dis 2: A13. PMC1323316 15670466

